# Assessment of the Post-Cracking Fatigue Behavior of Steel and Polyolefin Fiber-Reinforced Concrete

**DOI:** 10.3390/ma14227087

**Published:** 2021-11-22

**Authors:** Alejandro Enfedaque, Marcos G. Alberti, Jaime C. Gálvez, Jhonatan Santiago Proaño

**Affiliations:** 1 Departamento de Ingeniería Civil, Construcción, E.T.S de Ingenieros de Caminos, Canales y Puertos, Universidad Politécnica de Madrid, c/ Profesor Aranguren s/n, 28040 Madrid, Spain; alejandro.enfedaque@upm.es (A.E.); jaime.galvez@upm.es (J.C.G.); 2 Facultad de Ingeniería y Ciencias Aplicadas, Universidad Central de Ecuador, Av. América y Av. Universitaria, Quito 170129, Ecuador; nkt1010@hotmail.com

**Keywords:** fatigue, flexural fatigue, fiber-reinforced concrete, steel fiber, polyolefin fibers, concrete

## Abstract

Some types of fiber-reinforced concrete (FRC) such as steel fiber-reinforced concrete (SFRC) or polyolefin fiber-reinforced concrete (PFRC) are suitable for structural uses but there is still scarce knowledge regarding their flexural fatigue behavior. This study aimed to provide some insight into the matter by carrying out flexural fatigue tests in pre-cracked notched specimens that previously reached the Service Limit State (SLS) or the Ultimate Limit State (ULS). The fatigue cycles applied between 30% and 70% of the pre-crack load at 5 Hz until the collapse of the material or until 1,000,000 cycles were reached. The results showed that the fatigue life of PFRC both at SLS or ULS was remarkably higher than the correspondent of SFRC. The fracture surface analysis carried out found a linear relation between the fibers present in the fracture surface and the number of cycles that both SFRC and PFRC could bear.

## 1. Introduction

Fibre-reinforced concrete (FRC) appeared in the last century as an evolution of reinforced concrete (RC) as it solved some of the drawbacks that this material suffered when applied to structural elements. The development of a wide range of fibre types and their use in FRC provided a solution to shrinkage cracking and fire spalling, reduced the width of the cracks that appear in RC, and enabled the appearance of multifunctional concretes [1,2,3,4]. Moreover, some types of fibres such steel or polyolefin ones when forming steel fibre-reinforced concrete (SFRC) and polyolefin fibre-reinforced concrete (PFRC) have been capable of enhancing the mechanical properties of concrete to such extent that, according to some recommendations [5,6,7,8], the contribution of the fibres can be considered in the structural analysis [9,10,11].

Based on previous experiences, the use of FRC was expected to be beneficial in structural elements subjected to fatigue such as pavements, bridges, offshore platforms, railroad sleepers, or structures of factories with moving machinery [12,13]. When a FRC structural element is subjected to fatigue under compressive forces, the presence of fibres creates two opposed effects. First, fibres bridge the micro-cracks that appear and control the crack growth. Second, the presence of fibres increases the porosity, promoting the appearance of cracks, with the fibre dosage being a key parameter [14,15]. Moreover, the beneficial effect of fibres has been shown to be highly dependent on the fibre length, with the short fibres being the ones that produce the best results [16]. It should be underlined that in all these studies SFRC was employed.

Due to the recent development of polymer fibres that enhance the mechanical properties of plain concrete, on some occasions obtaining comparable properties to those of SFRC, the interest of assessing the fatigue behaviour of these type of concrete formulations has aroused. The study of this type of FRC, when subjected to fatigue cycles, is of key importance as there are previous studies where the structural character of such formulations has been assessed [17,18]. It has been shown that concrete reinforced with PVA fibres might boast better fatigue behaviour than certain SFRC formulations [19]. In concrete formulations with a cocktail of steel and PVA fibres, similar feature has been observed [20]. In any case, there is hardly any study where concretes with a certain dosage of macro polymer fibres have been subjected to fatigue cycles.

It should be highlighted that, in the mentioned applications, there are not only structural elements subjected to compressive fatigue but also to flexural fatigue. In these elements the reduction of stiffness, strength, toughness, or other properties that cyclic loads might cause could be even more dangerous for the structural integrity than in the case of compressed parts of the structure. Moreover, it should be taken into account that in such applications the most loaded sections of the structure might be cracked due to differential displacements or loads. Consequently, the research of this phenomenon in FRC elements is of great relevance, but up to now the amount of literature dealing with this matter is reduced. Due to the high difficulty of performing tensile tests in FRC specimens, most of the research has employed flexural tests. In one of them, the influence of the length of the fibres was assessed using hooked-end steel fibres [21]. It was found that the main reinforcement mechanism was based on the anchorage of the hook of the fibres, with the importance of the length of the fibre being negligible. However, additional contributions have shown that, for the same fibre dosage, shorter fibres were more effective to enhance the flexural fatigue behaviour of SFRC [22]. Regarding the shape of the fibres, it was shown that the hooked-end fibres provided a greater improvement of the mechanical behaviour of concrete than the straight ones [23]. It should be underlined that all the mentioned studies were carried out with SFRC, with, in the opinion of the authors, hardly any study where macro-polymer structural fibres were employed.

As was seen in previous studies, the behaviour of plain concrete elements when subjected to fatigue depends on several parameters such as the loading conditions, frequency, number of cycles, and concrete mix design, among others. To the mentioned parameters, some additional ones, such as the type of fibre, fibre dosage, distribution and orientation, and pre-crack width, should be included when FRC is studied.

Additionally, there are few studies that dealt with the behaviour of concrete reinforced with polymer fibres. Regarding the compressive fatigue behaviour, [24] conducted a study where it was shown that fibres improved the material performance when subjected to low frequencies’ loading. Such improvement was explained by the effect of fibres bridging the cracks generated during the tests. In another study, where micro-polypropylene fibres were added to a concrete that was subjected to cyclic loading, it was shown that also micro-fibres enhanced the compressive fatigue behaviour of plain concrete [25]. Other authors have studied the flexural fatigue behaviour of concrete reinforced with micro polymer fibres in unnotched specimens [26]. In such a study it could be seen that the presence of fibres increased the number of cycles that the material could bear. Regarding other studies performed in unnotched specimens where macro polymer fibres were used for reinforcing concrete subjected to flexural fatigue, in [27] it was evident that volume fractions of polypropylene fibres equal to 0.5%, 1.0%, and 1.5% improved the fatigue lives of plain concrete but also introduced a greater variability. As it was mentioned, there are a certain number of studies that dealt with the flexural fatigue of concretes reinforced with polymer fibres; nevertheless, it cannot be overlooked that there are few of them that considered the possibility of the material being pre-cracked [28].

Consequently, and seeking to improve the current state of the art regarding the fatigue behaviour of FRC, this study carried out an experimental campaign in two formulations of SFRC and PFRC with similar fibre volume fractions, seeking to assess the flexural fatigue behaviour of PFRC and compare their fatigue flexural behaviour. In addition, the influence of the pre-crack width in the behaviour of SFRC was determined. Obtaining results in these three aspects will significantly increase the reliability of the use of SFRC and PFRC when employed in structures where fatigue loading conditions are relevant. Moreover, the comparison of the fatigue behaviour of SFRC and PFRC will provide another parameter for choosing the most apt FRC in structural applications.

## 2. Material Production and Preparation

**Figure 1:**
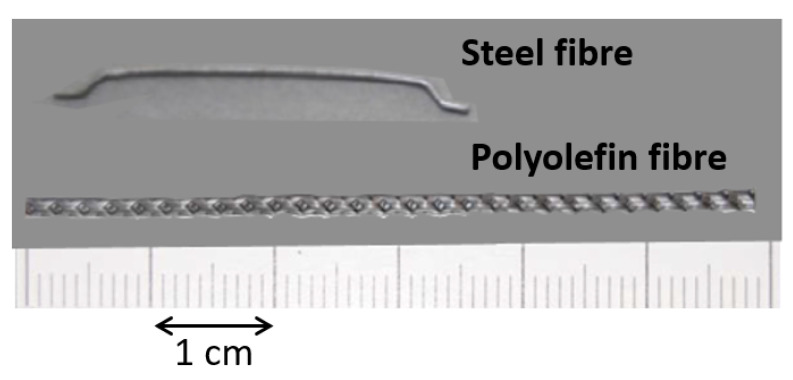
Appearance of the fibres used.

In order to perform the present research, two formulations of concrete were used. The first formulation was a conventional concrete, termed VCC-PFRC4.5, which included 4.5 kg/m^3^ of polyolefin fibres. The second formulation was a self-compacting concrete, called SCC-SFRC50, where 50 kg/m^3^ of hooked-end steel fibres were added. VCC-PFRC4.5 and SCC-SFRC50 boasted a volume fraction of fibres of 0.49% and 0.64%, respectively. The characteristics of the steel and polyolefin fibres can be seen in Table 1. Similarly, the outlook of such fibres can be seen in Figure 1.

The cement used in both formulations was EN 197-1 CEM I 52.5 R-SR 5. Limestone powder was employed as a micro-aggregate, having a specific gravity of 2700 kg/m^3^ and a Blaine surface of 425 m^2^/kg. This material boasted high purity, as its calcium carbonate content was greater than 98%. In order to obtain the required viscosity, a superplasticiser based on polycarboxylates was employed. Regarding the characteristics of the aggregates, it should be mentioned that the gravel (12–4 mm), grit (8–4 mm), and sand (0–2 mm) were siliceous; the maximum and minimum aggregate sizes can be seen in parenthesis, respectively.

Both concrete formulations were manufactured in a planetary mixer with a maximum capacity of 100l at laboratory conditions. Table 2 shows the mix proportioning used in VCC-PFRC4.5 and SCC-SFRC50.

Using each type of concrete, six specimens of 430 × 100 × 100 mm^3^ were performed. Regarding the pouring and consolidation methods, it should be highlighted that, in the case of SCC-SFRC50, the moulds were filled from one of the sides and concrete was levelled by the action of its own weight. In the case of VCC-PFRC4.5, the moulds were filled in their sides and, afterwards, in the middle zone of the mould. Then, the specimens were consolidated during 10 s by using a vibratory table [29,30]. As the fibres were added to the mixer during the production process, they were randomly distributed in the fresh concrete before being poured. Nevertheless, as the different pouring and consolidating methods employed in VCC-PFRC4.5 and SCC-SFRC50 might have influenced the positioning of fibres, an analysis of the fracture surfaces generated in the fatigue tests was performed, as shown in following sections. After the production process, all specimens were stored in a climatic chamber at 20 °C and above 95% of relative humidity until testing. Cylindric specimens were manufactured in order to obtain the mechanical properties of the concrete mixes. While the mechanical properties were obtained at 28 days of age, the fatigue tests were performed when the specimens were 55 months old. An extended explanation of the production processes and the mechanical characterisation of the formulations can be found in [31].

**Figure 2:**
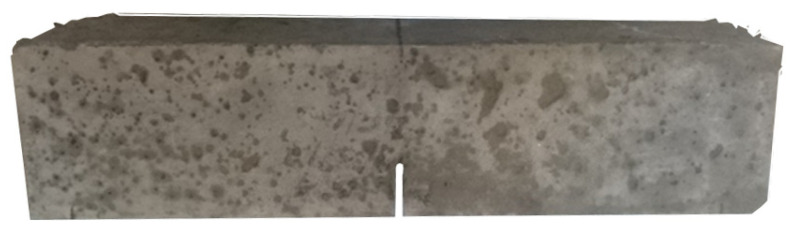
Appearance of the notched prismatic specimens before testing.

The prismatic specimens were prepared for the flexural fatigue tests according to RILEM TC-187 [32]. A notch of a third of the ligament depth was performed on the central section of the prismatic specimens. In order to avoid introducing any damage in the material, the notches were carried out by using a water-cooled circular saw equipped with a diamond disc. An image of one of the specimens can be seen in Figure 2.

## 3. Test Setup

**Figure 3:**
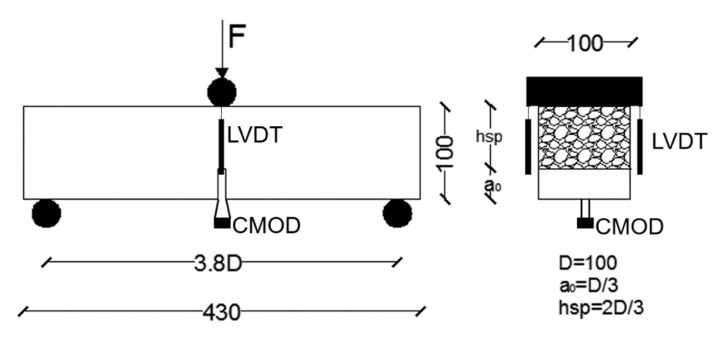
Test setup. Measures in mm.

The test setup followed [24] in its main characteristics. However, the span between the bearing cylinders that is usually 4*D* was reduced to 3.8*D* to avoid instabilities during the fatigue cycles. Reducing the span between the bearing cylinders implies a certain increment in the load registered during the tests. The loading cylinder was placed in the middle section of the specimens, right above the tip of the notch. A sketch of the test setup and some of the gauges used in the test can be seen in Figure 3.

**Figure 4:**
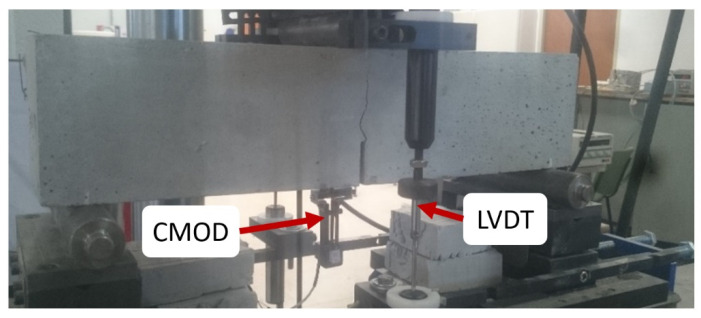
Image of a specimen while testing.

The tests were carried out with an Instron 8803 testing machine (Instron, Buckkinghamshire, UK) of 500-kN capacity. The crack mouth opening displacement (CMOD) was measured by means of a clip-on gauge device attached to the lips of the notch. Regarding the deflection, two linear variable differential transformers (LVDT) were placed at both sides of the notch of the tested specimen. During the tests, the following data were registered: position of the actuator, the applied load, the CMOD opening, and the deflection at both sides of the sample. An image of a specimen while testing can be seen in Figure 4.

Flexural fatigue tests were performed on pre-cracked specimens that had been previously tested under quasi-static conditions. Quasi-static tests were controlled by the displacement rate of the actuator setting three different stages. The first two stages corresponded to loading stages, being the third an unloading one. During the first stage, the actuator moved at 0.0425 mm/min until reaching 6 mm of displacement. In the second stage, the rate increased up to 0.17 mm/min until the desired crack width was reached. Two crack widths were chosen. In order to assess the residual bearing capacity under fatigue, in the test of the elements that had reached the Ultimate Limit State (ULS) according to [24], the displacement of the quasi-static test was stopped when a CMOD value of 2.5 mm was reached. In the mentioned recommendation, such opening is often referred as *f_R3_*. The residual bearing capacity under flexural fatigue when specimens reached a cracking state compatible with the Service Limit State (SLS) that corresponded to a CMOD of 0.5 mm was also determined. Such a point is referred to in [24] as *f_R1_*. For each specimen the load borne at the correspondent *f_Ri_* value was recorded, as it will be the reference value for the flexural fatigue tests.

**Figure 5:**
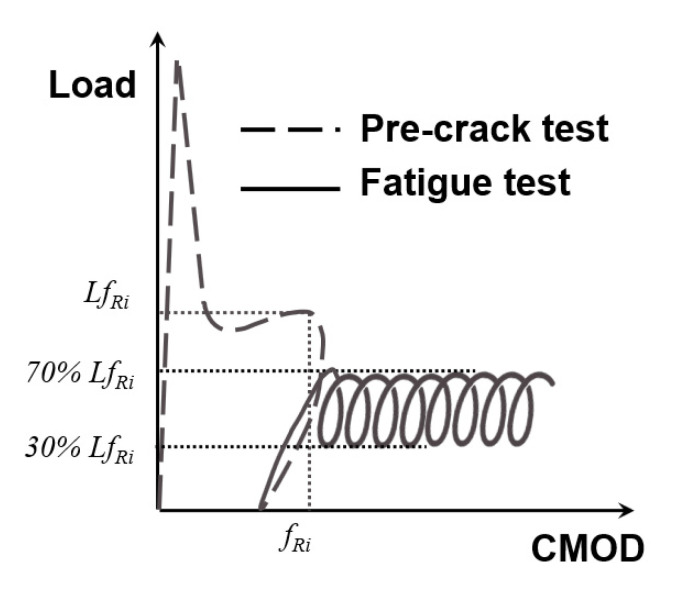
Sketch of the test procedure that included both the pre-crack test and the fatigue test.

The fatigue tests adopted the configuration shown in Figure 3 and Figure 4. The test was divided into two stages. In the first stage, the load increased up to 0.5 of the load at the *f_Ri_* value chosen (*Lf_Ri_*). The maximum and minimum loads of the tests were constant during the tests, being equal to 0.7*Lf_Ri_* and 0.3*Lf_Ri_*, respectively. Such values define an amplitude (0.7*Lf_Ri_*/0.3*Lf_Ri_*) of 0.43, which was constant during all tests. In order to reduce the number of parameters studied, the frequency was kept constant at 5 Hz. The definition of the loading parameters was based on previous studies [33,34,35]. A sketch of the test stages can be seen in Figure 5.

In order to determine the possible causes of scattering, a counting exercise regarding the fibres present in the ligament section was carried out following the methodology presented in [36,37]

## 4. Results

The formulations were tested in order to obtain their main mechanical properties, such as compressive strength, indirect tensile strength, and modulus of elasticity. Such tests were performed according to the following recommendations: EN 12390-3:2009 [38] (compressive strength), EN 12390-6:2009 [39] (indirect tensile strength), and EN 12390-13 [40] (modulus of elasticity). Table 3 shows the mechanical properties of all the formulations performed. No sound remarks were found regarding the influence of the properties shown in Table 3 and the results and conclusions obtained in the fatigue tests.

**Figure 6:**
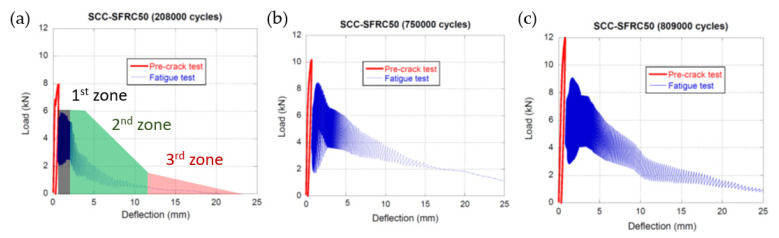
Fatigue behaviour of SCC-SFRC50 specimens pre-cracked at *f_R1_*: (**a**) specimen collapsed after approximately 208,000 cycles, (**b**) specimen collapsed after approximately 750,000 cycles, and (**c**) specimen collapsed after approximately 809,000 cycles.

The results of the fatigue tests performed on the SCC-SFRC50 specimens that had been pre-cracked up to *f_R1_* can be seen in Figure 6. Each valid test can be seen in an individual plot. In each plot appears one red curve that corresponds to the fracture test performed in order to reach the SLS (when the CMOD was equal to 0.5mm). Moreover, in blue, one can see the behaviour of material when subjected to the fatigue tests under the conditions previously mentioned in Section 3. In every plot, it is easy to perceive three zones that corresponded to three different processes in the material. The first zone is right next to the fracture curve and shows how the material was able to withstand the loads imposed by the fatigue cycles without suffering a notorious damage. In this zone the cycles were not easy to perceive as there were no noticeable changes in the deflection and, consequently, some of the cycles might be superimposed. The second zone can be described as the part where the material begins to lose its load-bearing capacity and the amplitude of the loads imposed was reduced. In this part of the curves, the deflection increased in a steady and low rate, while the load-bearing capacity decreased. The third zone corresponded to the part of the curves where the material was not able to cope with the cyclic loads imposed and the appearance of the fatigue load-deflection curve had a remarkable resemblance to the load-deflection curve obtained in a fracture test. It should be underlined that the difference in the fatigue cycles borne by the specimens was located in the initial part of the curves, with the two final zones of the curves alike, at least in the specimens that sustained 750,000 and 809,000 cycles. Another feature that should be mentioned is that the maximum load values of each cycle described a curve quite similar to the one that can be obtained in a static fracture test.

**Figure 7:**
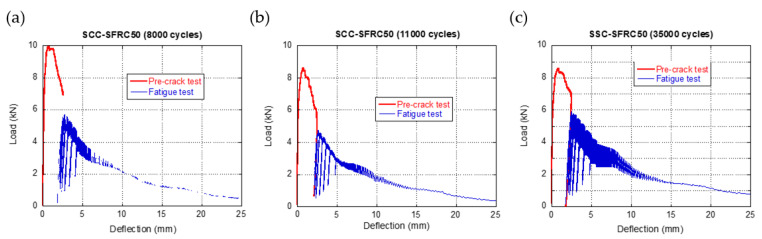
Fatigue behaviour of SCC-SFRC50 specimens pre-cracked at *f_R3_*: (**a**) specimen collapsed after approximately 8000 cycles, (**b**) specimen collapsed after approximately 11,000 cycles, and (**c**) specimen collapsed after approximately 35,000 cycles.

When the specimens were pre-cracked until the ULS was reached (*f_R3_*, which corresponded to a crack width of 2.5 mm) and then fatigue cycles, with the same characteristics previously mentioned, were imposed, the curves that can be observed in Figure 7 were acquired. Similarly to the curves of Figure 6, the red curves correspond to the fracture tests and the blue ones to the fatigue tests. Regarding the fracture behaviour of the material, it can be clearly seen that the initial zone of the curves that appear in Figure 6 does not appear in Figure 7. On the contrary, the second part of the curves, where the increment of the deflection and the reduction of the load-bearing capacity are observed, it can be clearly noticed following the fracture curve. Additionally, the final part of the curves, where the behaviour of the material was similar to that obtained in a static fracture test, is also perceivable. Analogously to what was explained regarding the curves of Figure 6, the difference in the cycles supported by the specimens seemed to be in the number of cycles that did not generate a clear damage in the material.

**Figure 8:**
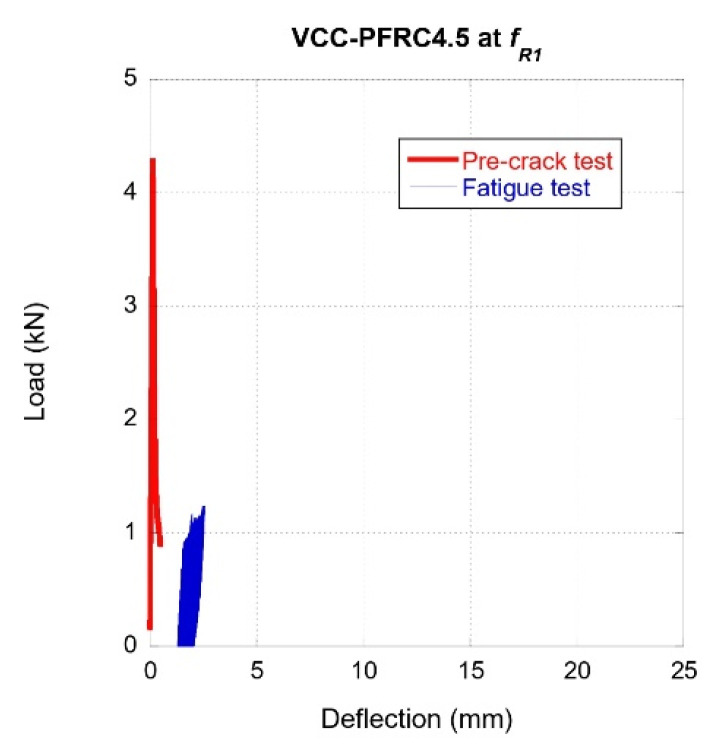
Fatigue behaviour of VCC-PFRC4.5 specimens pre-cracked at *f_R1._*

VCC-PFRC4.5 specimens were subjected to fatigue tests after carrying out a pre-crack that corresponded to a SLS (*f_R1_*, which corresponded to a crack width of 0.5 mm). The results of such tests can be observed in Figure 8. The characteristics of the load cycles were quite similar to the ones applied to the SCC-SFRC50 specimens. However, the specimens manufactured with VCC-PFRC4.5 were able to withstand up to 1,000,000 cycles without collapsing or suffering remarkable damage. Reaching 1,000,000 cycles without collapsing was considered the endurance of the material. As can be seen in Figure 8, although a certain increment of the deflection is perceived, its values remained in within the limits established for calculating the ULS. In addition, no remarkable changes in the stiffness of the material can be noticed.

**Figure 9:**
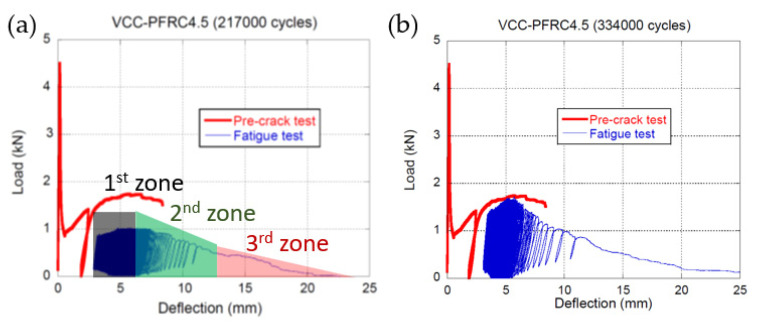
Fatigue behaviour of VCC-PFRC4.5 specimens pre-cracked at *f_R3_*: (**a**) specimen collapsed after approximately 217,000 cycles and (**b**) specimen collapsed after approximately 334,000 cycles.

In Figure 9 the curves obtained in the fracture, up to ***f_R3_***, and fatigue tests performed on VCC-PFRC4.5 specimens can be seen. Similarly to what is seen in Figure 6, the fatigue behaviour seemed to feature the same three zones previously mentioned. Moreover, the shape of the envelope of the maximum load of each cycle clearly resembles the shape obtained in a static fracture test of the material. However, it seemed that the load values were somewhat lower than those obtained in the fracture test. This can be partially explained as the loads that defined the cycles were only a certain percentage of the ones that the material could sustain in a static fracture test.

In Table 4 a summary of the fatigue test results can be seen. In this table it can be clearly perceived that, after reaching the SLS or the ULS alike, if a structural member was subjected to fatigue cycles, a greater reliability was obtained if such a member was manufactured with VCC-PFRC4.5 if compared with SCC-SFRC50.

## 5. Discussion

**Figure 10:**
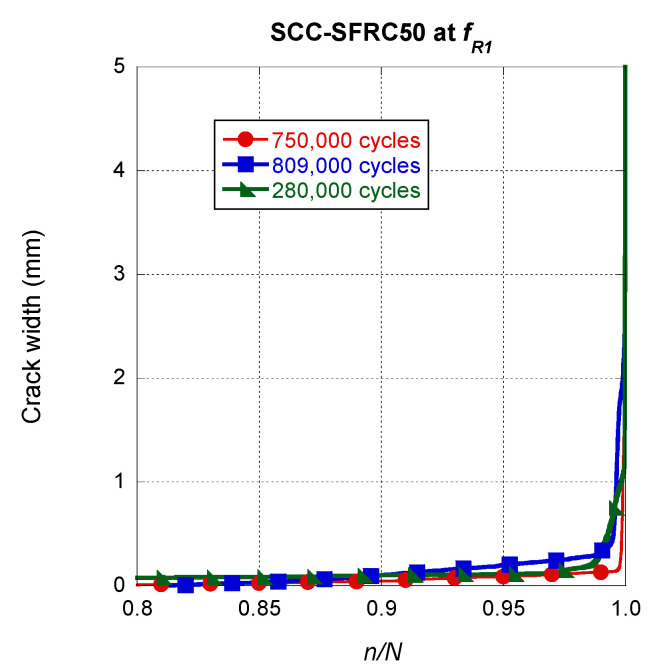
Crack width evolution comparison among the SCC-SFRC50 specimens pre-cracked at *f_R1._*

The scattering in the fatigue tests was studied for both formulations in which specimens collapsed. In the case of SCC-SFRC50 specimens that had been pre-cracked at *f_R1,_* we observed a great discrepancy in one of the three tests conducted. In such a test, only 280,000 cycles could be performed, whereas in the other two, around 800,000 were achieved. In order to analyse the potential differences in the cracking process among the specimens tested, Figure 10 was performed. Moreover, a counting exercise was carried out to detect if there was any relation between the number of fibres in the fracture surfaces and the number of cycles that the specimens were able to sustain.

The influence of the number of cycles was reduced by dividing the cycle *n* by the number *N* of cycles that the specimen was able to sustain before collapsing *N*. By doing this, the curves of all tests could be compared. In Figure 10, it can be seen that in all specimens there was a reduced, but steady, growth of the crack width as the number of cycles increased. It should be mentioned that the slope in such curves before the final fracture appeared was similar in all specimens. Additionally, the appearance and progress of the final fracture seemed to be similar no matter the number of cycles that the specimen was capable of bearing.

**Figure 11:**
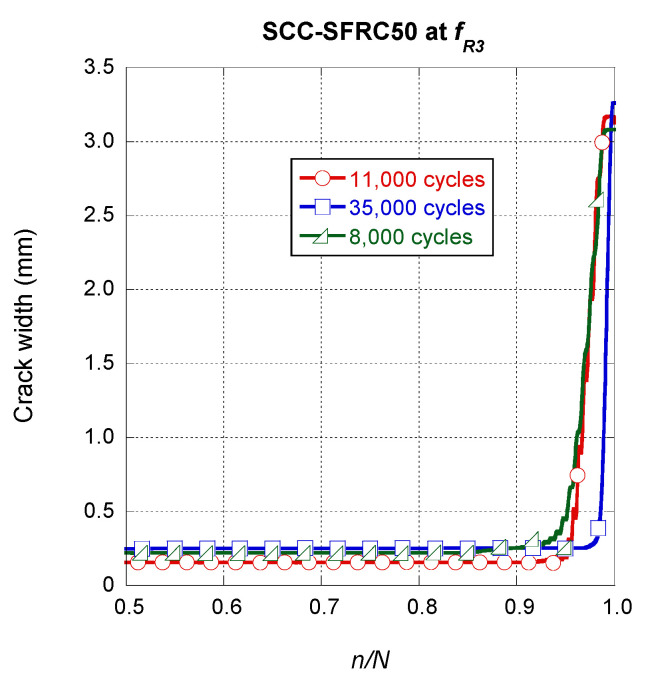
Crack width evolution comparison among the SCC-SFRC50 specimens pre-cracked at *f_R3._*

If a similar analysis was performed on the SCC-SFRC50 specimens pre-cracked at *f_R3_*, it could be seen that there were no remarkable differences among them. As shown in Figure 11, at this scale, the steady growth that the crack width of the specimens underwent can be hardly perceived. Once the growth of the final fracture began, slightly above a n/N value of 0.9, the shapes of the curves had a great resemblance. Nevertheless, it should be mentioned that in the case of the specimen that was able to sustain a greater number of cycles, the growth of the final fracture appeared at a slightly greater value of *n/N*.

**Figure 12:**
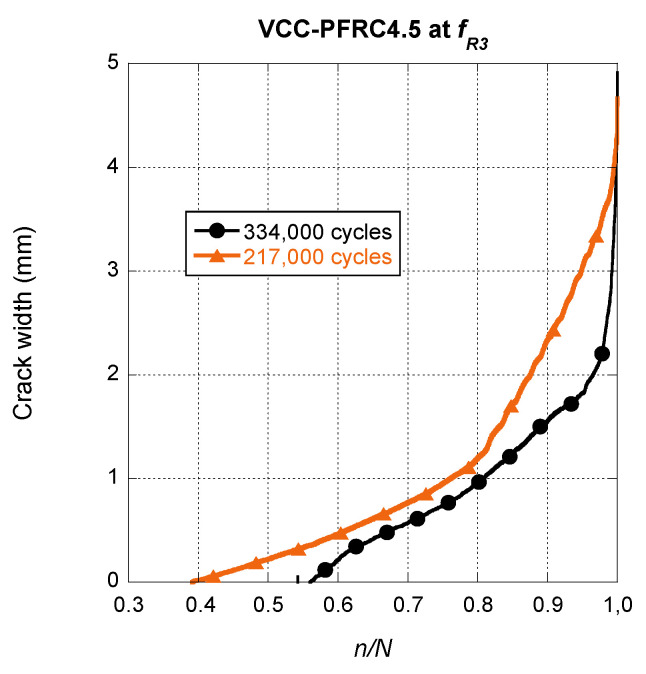
Crack width evolution comparison among the VCC-PFRC4.5 specimens pre-cracked at *f_R3._*

Regarding the crack width evolution in the VCC-PFRC4.5 specimens, a completely different tendency can be observed in Figure 12.

Observing Figure 12, it should be mentioned that the shape that displayed any of the crack width evolution curves of VCC-PFRC4.5 was remarkably different from the ones displayed for SCC-SFRC50, both for the *f_R1_* and *f_R3_* cases. The steady growth part of the curve is not clearly noticed and is difficult to detect where the growth of the final fracture appears. This phenomenon can be perceived in the case of the specimen that collapsed at 217,000 cycles, being not so evident in the case of the other specimen. Another point that is worth to mention is that the onset of the steady growth phase was between a *n/N* value of 0.4 and 0.55.

**Figure 13:**
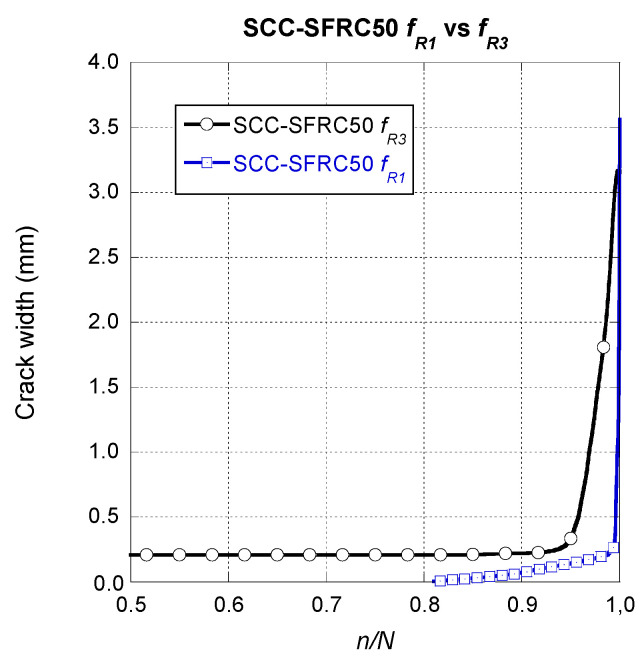
Crack width evolution comparison among the SCC-SFRC50 specimens pre-cracked at *f_R1_* and *f_R3._*

In order to determine the influence of the pre-crack width in the residual fatigue load-bearing capacity of SCC-SFRC50, a comparison of the average curves shown in Figure 10 and Figure 11 was carried out. Such curves can be seen in Figure 13. It can be seen that in the case of the specimens pre-cracked at *f_R1_* the growth of the crack width is remarkably steadier than in the case of the specimens pre-cracked at *f_R3_*. This is of a great importance because it supposes that when ULS is reached in a structural member without any other reinforcement the propagation of damage if fatigue cycles are introduced is sudden and develops rapidly. On the contrary, when SLS is applied in a SCC-SFRC50 structural element and later fatigue cycles are applied, the crack growth onsets right after 80% of the endurance of the material is surpassed. Moreover, the crack growth is progressive almost until the total endurance of the material is reached.

According to the data that can be seen in Table 4, it can be stated that the average number of fatigue cycles that a SCC-SFRC50 specimen can sustain when ULS is reached was only 2.94% of the number of cycles resisted at SLS. If a comparison between the fatigue life expectancy of SCC-SFRC50 and VCC-PFRC4.5 at ULS is performed, it can be stated that the one of SCC-SFRC50 is on average only 5.14% of the correspondent of VCC-PFRC4.5.

**Figure 14:**
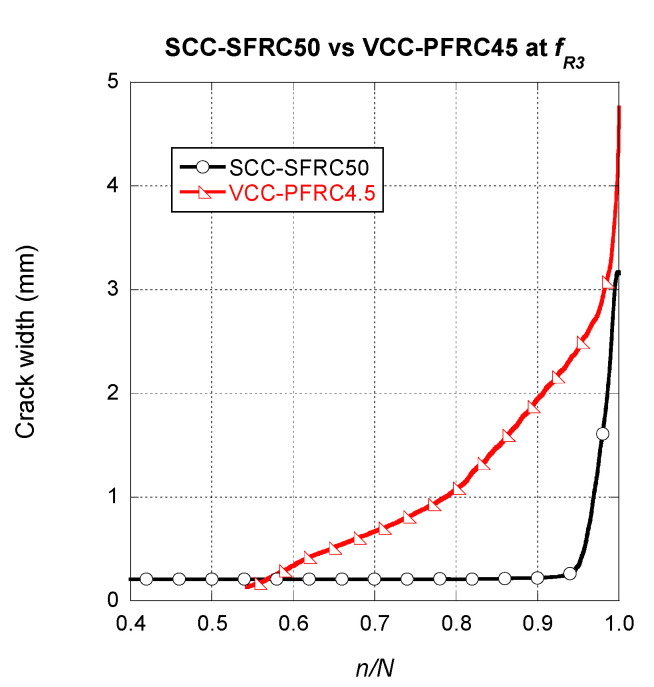
Crack width evolution comparison among the SCC-SFRC50 specimens and the VCC-PFRC4.5 pre-cracked at *f_R3._*

Another point that should be compared is the behaviour under cyclic loads of structural members manufactured with SCC-SFRC50 and VCC-PFRC4.5 that have reached the ULS. To perform such a comparison, the average behaviour of the correspondent formulations is plotted in Figure 14. While in the case of the SCC-SFRC50 curve there is a part of the curve where a steady increase of the crack width and later a progression of the fracture process appears, when dealing with VCC-PFRC4.5 such two parts are not clearly perceived. This phenomenon was noticed because the slope of the steady growth part was notably greater than in the SCC-SFRC50 curves. In addition, the slope of the curve in the final fracture process was remarkably lower than in the SCC-SFRC50 curve. Consequently, the change in the slope was much more moderated and these two parts were more difficult to notice. Another feature that should be highlighted is that the steady progress of the crack width was only obtained with the VCC-PFRC4.5 formulation. This characteristic is directly related to the presence of the polyolefin fibres. Thus, with the use of the concrete formulation with an addition of polyolefin fibres, a greater safety is obtained if the ULS is reached when it was compared with SFRC.

**Figure 15:**
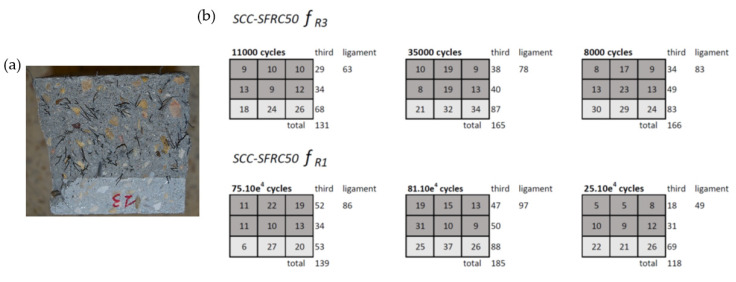
(**a**) Appearance of the fracture surface of a SCC-SFRC50 specimen, (**b**) fracture surface analysis.

**Figure 16:**
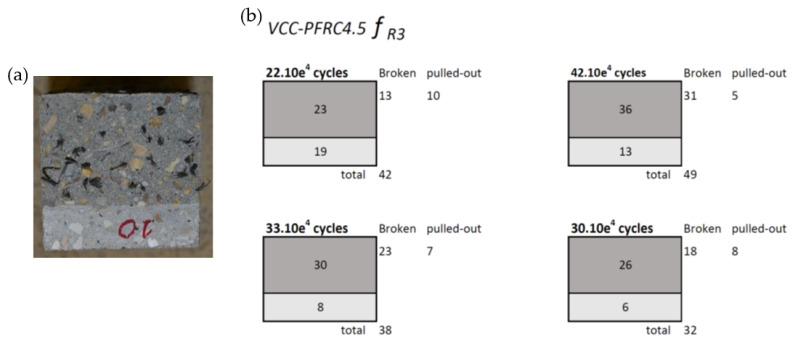
(**a**) Appearance of the fracture surface of a VCC-PFRC4.5 specimen. (**b**) Fracture surface analysis.

A fracture surface analysis was carried out to assess the cause of the scattering obtained in the tests. Not only the number of fibres in the fracture surfaced was determined, but also their positioning within the fracture surface was registered. Figure 15 shows the steel fibres found in each fracture surface of SCC-SFRC50. In addition, the fracture surface was divided into nine sectors and the number of fibres present in such sectors was found. Regarding VCC-PFRC4.5, the number of fibres located in the ligament and the notch can be observed in Figure 16. Moreover, the number of broken and pulled-out fibres was also determined.

Comparing the results shown in Table 4 and the fracture surface analysis that can be seen in Figure 15, the discrepancies in the fatigue behaviour of the SCC-SFRC50 specimens pre-cracked at *f_R1_* can be explained. The fibres present in the specimen that was able to sustain around 208,000 cycles had approximately 50% of the fibres present in the fracture surface of the other two specimens. However, if the number of cycles sustained are compared, such a specimen only sustained around 25% of the cycles borne by the other two specimens. This phenomenon might imply that the damage introduced in the pre-crack test was remarkably higher in the specimen with a fewer number of fibres. In the case of the VCC-PFRC4.5 specimens, it was not possible to perform an analogue analysis because there were no notable discrepancies among the test results.

**Figure 17:**
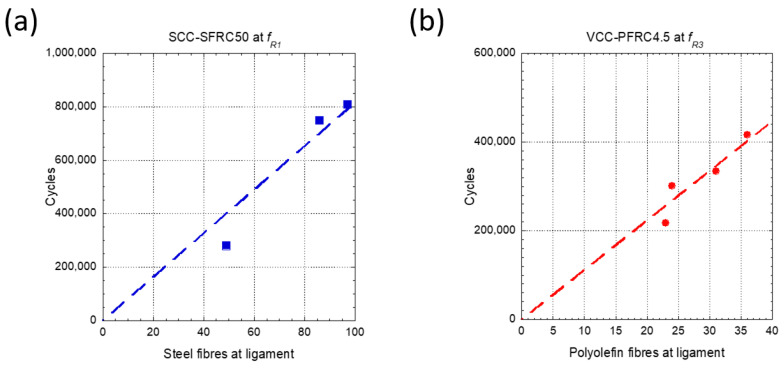
(**a**) Relation between the number of fibres in the ligament of SCC-SFRC50 pre-cracked at ULS and the number of cycles before failure. (**b**) Relation between the number of fibres in the ligament of VCC-PFRC4.5 pre-cracked at SLS and the number of cycles before failure.

As can be seen in Figure 17a, there was a linear relation between the number of fibres present in the ligament of the SCC-SFRC50 specimens pre-cracked at *f_R1_* and the number of cycles that the specimens were able to sustain before collapsing. On the contrary, no relation could be found between the number of cycles before failure of the SCC-SFRC50 specimens pre-cracked at *f_R3_* and the number of fibres of the ligament, central third, or the complete section. This phenomenon might imply that when reaching ULS, which corresponded to a crack width of *f_R3_*, the improvement that the steel fibre provided to the material was heavily consumed.

As can be seen in Figure 17b, there was a linear relation between the number of fibres in the ligament of the VCC-PFRC4.5 specimens pre-cracked at *f_R3_* and the cycles that such specimens could bear before collapsing. In the case of the specimens of such formulation pre-cracked at *f_R1,_* no relation could be established as the fatigue tests ended when reaching 1,000,000 cycles The discrepancies in the behavior of SCC-SRFC50 and VCC-PFRC4.5 could be attributed to the different mechanical properties of the fibers and fiber-matrix anchorage mechanisms. Consequently, while the polyolefin fibers were capable of changing the FRC behavior both at *f_R1_* and *f_R3,_* steel fiber seemed to be more relevant at *f_R1_*.

## 6. Conclusions

Steel and polyolefin fibre-reinforced concrete specimens were subjected to fatigue cycles after reaching the crack width that corresponded to SLS and ULS. The fatigue cycles were defined to apply loads from 30% to 70% of the load registered at SLS or ULS, respectively, with a frequency of 5 Hz. For all the pre-crack widths and formulations, it was observed that the shape of the load-deflection curves resembled the shape that could be obtained in fracture tests. It is important to highlight that no sudden or brittle collapse appeared in any of the tests performed. Consequently, FRC seemed to be a reliable and suitable material for structural applications where cyclic loads play a significant role.

The results of the fatigue tests applied to VCC-PFRC4.5 showed that pre-cracked specimens at SLS did not collapse after 1,000,000 cycles. Nevertheless, it should be mentioned that, as the crack width increased, certain damage was inflicted in such specimens. However, the crack width did not reach the values that corresponded to ULS. In the case of the SCC-SFRC50 specimens pre-cracked at *f_R1_*, all of them collapsed before reaching 1,000,000 cycles. Regarding the test results that were carried out after pre-cracking the specimens at ULS, it should be mentioned that the number of fatigue cycles that the specimens suffered before collapsing was remarkably higher in the case of VCC-PFRC4.5 than in the case of SCC-SFRC50. The differences in the behaviour of the materials noticed in the tests supposed that VCC-PFRC4.5 provides a greater reliability than SCC-SFRC50 for the case of structural members subjected to fatigue cycles after reaching SLS and ULS alike. Thus, it seems that the addition of a certain dosage of polyolefin fibres provides greater reliability than the addition of a similar volume fraction of steel fibres.

When a specimen of SCC-SFRC50 reached the ULS, the evolution of the crack width as the cycles progressed was limited to the last 10% of the cycles, having hardly any steady growth. In contrast, if such a specimen had only reached SLS, the steady growth of the crack almost covered the last 20% of the cycles. Regarding VCC-PFRC4.5 at ULS, it can be stated that once past the 50% of the cycles the crack growth began and continued almost until the fracture of the specimen.

After the counting exercise was performed it can be stated that in the case of the VCC-PFRC4.5 formulation there was a linear relation between the number of fibres present in the fracture surface and the endurance of the specimens pre-cracked at *f_R3_*. No correlation could be found in the case of the tests carried out in VCC-PFRC4.5 specimens pre-cracked at *f_R1_* because such specimens withstood 1,000,000 cycles without collapsing. Regarding SCC-SFRC50, there was also a linear relation between the number of fibres in the fracture surface and the number of cycles before the failure of the specimens pre-cracked at SLS. In the specimens of SCC-SFRC50, where ULS was reached, it seemed that the bridging action of the fibres was heavily damaged because there was no relation between the number of fibres in the fracture surface, the central third, or the complete section.

## Figures and Tables

**Table 1:** Properties of the fibres employed.

Property	Polyolefin Fibers	Steel Fibers
Density (kg/m^3^)	910	7850
Length (mm)	60	35
Equivalent diameter (mm)	0.903	0.550
Tensile strength (MPa)	400	1100
Modulus of elasticity (GPa)	9	210
Fibers per kg	27,000	14,500
Ultimate strain (%)	20	8

**Table 2:** Concrete formulation per m^3^.

Property	VCC-PFRC4.5	SCC-SFRC50
Cement (kg/m^3^)	375	425
Limestone powder (kg/m^3^)	100	210
Water (kg/m^3^)	187.5	199
Sand (kg/m^3^)	916	947
Gravel (kg/m^3^)	450	492
Grit (kg/m^3^)	300	-
Superplasticizer (% relative to cement weight)	0.75	0.72

**Table 3:** Mechanical properties of the concrete mixes. Coefficient of variation shown in parenthesis.

Property	VCC-PFRC4.5	SCC-SFRC50
Modulus of elasticity (GPa)	31 (0.07)	34.1 (1.48)
Compressive strength (MPa)	31.3 (0.07)	52.2 (1.23)
Tensile strength (MPa)	4.24 (0.13)	7.8 (4.49)

**Table 4:** Number of load cycles when the specimens collapsed. Fatigue tests were stopped at 1,000,000 cycles. The pre-crack width is stated in the table (SLS or ULS).

Service Limit State (*f_R1_*)		Ultimate Limit State (*f_R3_*)	
VCC-PFRC4.5	SCC-SFRC50	VCC-PFRC4.5	SCC-SFRC50
>1,000,000	≈750,000	≈416,000	≈11,000
>1,000,000	≈809,000	≈334,000	≈8000
-	≈208,000	≈301,000	≈35,000

## Data Availability

Data is contained within the article.
